# Cross-scale design of chemosensor arrays: from molecular self-assembly in water to paper-based devices for metal ion detection

**DOI:** 10.3762/bjnano.17.59

**Published:** 2026-06-24

**Authors:** Yui Sasaki, Tsuyoshi Minami

**Affiliations:** 1 Research Center for Advanced Science and Technology, The University of Tokyo, 4-6-1, Komaba, Meguro-ku, Tokyo 153-8904, Japan; 2 JST PRESTO, 4-1-8 Honcho, Kawaguchi, Saitama 332-0012, Japan,; 3 Institute of Industrial Science, The University of Tokyo, 4-6-1 Komaba, Meguro-ku, Tokyo 153-8505, Japan

**Keywords:** chemosensor, metal ion, paper device, pattern recognition, self-assembly

## Abstract

Monitoring metal ions in aquatic environments is essential for understanding ecosystem dynamics and assessing water quality. Chemosensors have emerged as powerful analytical tools at the molecular level that transduce ion-recognition events into optical signals. In particular, coordination with metal ions enables cross-reactive sensing, allowing for simultaneous detection of multiple analytes based on pattern recognition. This perspective summarizes advances in self-assembled chemosensor systems for metal ion detection and further developments to solid-state chemosensor array devices based on nanotechnologies. In practical chemosensor designs, dynamic covalent bonds between catechol and phenylboronic acid derivatives have been employed to provide a versatile strategy for the spontaneous preparation of chemosensor elements without extensive synthetic effort. Furthermore, integration of these self-assembled chemosensors into paper substrates enables portable detection platforms for on-site analysis without using stationary laboratory instruments. This cross-scale design strategy provides a promising approach for simultaneous detection of metal ions in various water environments across microscopic and macroscopic scales.

## Introduction

Metal ions are essential species in biological systems; however, heavy metal ions act as pollutants and can damage organs and the immune system through the intake of contaminated food [1–2]. Globally, the monitoring of metal ion levels in water environments is significant.

Chemosensors are analytical tools at the molecular level that exhibit optical responses through colorimetric and fluorescence signals [3–5]; they have been widely applied to metal ion detection [6–11]. The detection principle of chemosensors relies on ion and molecular recognition chemistry [3]. Target metal ions have various sizes and valencies; thus, simultaneous detection of multiple metal ions is desired [1–2]. In this regard, chemosensors provide multivalent interactions toward analytes, which allow the simultaneous detection of metal ions based on pattern recognition [12–16].

Chemosensor design relies on molecular-recognition chemistry, dye chemistry, and photochemistry [3]. Conventional chemosensors based on covalent bond chemistry require organic synthetic approaches in the chemosensor preparations [6–9]. Meanwhile, molecular self-assembly serves as a driving force for easy-to-obtain chemosensors through spontaneous preparation without a synthetic burden [17]. Moreover, the optical properties of chemosensors are tuned during association and dissociation upon analyte detection [18–19]. The performance of chemosensors has been widely evaluated in the solution state using conventional spectroscopic instruments [12–16], whereas the practical implementation of sensing systems remains challenging in on-site analysis [20–21]. Thus, the development of a solid-state chemosensor device is necessary for facile analyte detection.

To implement the cross-scale design concept, chemosensor elements should be highly dispersed on solid support materials possessing nanoarchitectures such as fiber structures. Paper substrates, which are commonly used in daily life, are eco-friendly and highly processable materials for disposable analytical devices [20–25]. In particular, the fiber structures of paper substrates provide unique functional nanoarchitectures [26–27]. Regarding analytical devices, such fiber-like nanostructures contribute to the homogeneous dispersion of chemosensor molecules through office printing technologies [28–29]. The printed chemosensor arrays on a paper substrate exhibit optical changes upon analyte capture, which are detected using portable recording apparatuses (e.g., smartphones, digital cameras, and flatbed scanners) [21,30]. Approaches using paper substrates as solid supports for chemosensor array devices enable on-site analysis without using stationary laboratory facilities.

**Figure 1:**
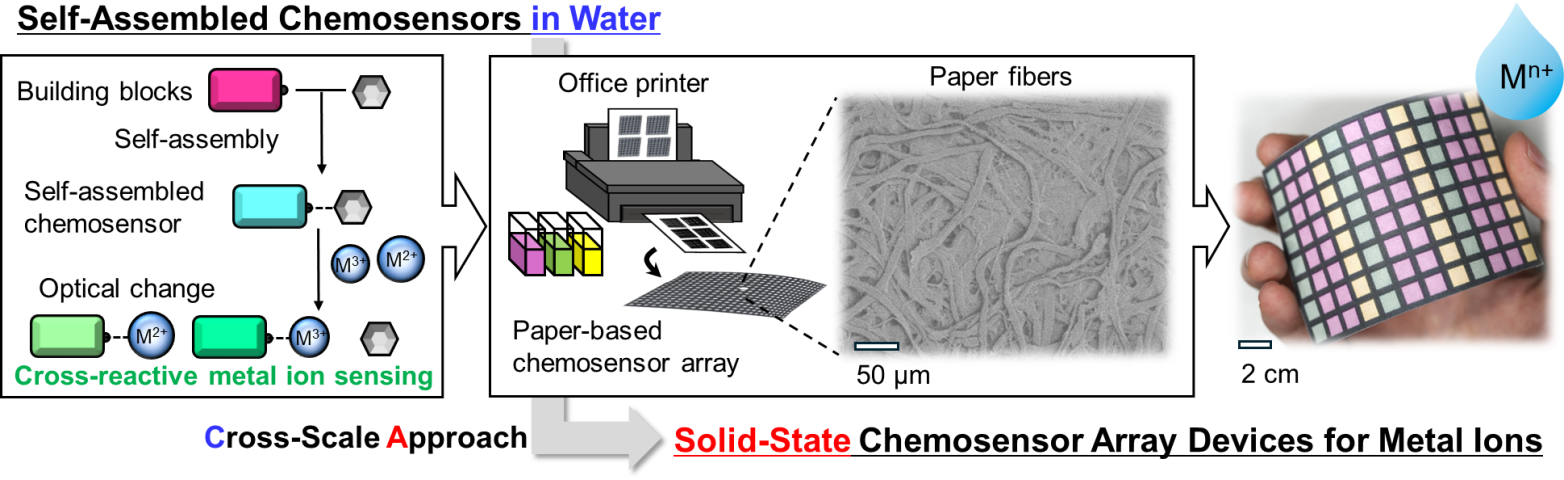
Conceptual figure showing this perspective on simultaneous metal ion detection based on self-assembled chemosensors and their devices through a cross-scale design. The middle panel of Figure 1 was adapted from [28] (“Nanoarchitectonics of highly dispersed polythiophene on paper for accurate quantitative detection of metal ions”, © 2024 Y. Sasaki et al., published by the Royal Society of Chemistry, distributed under the terms of the Creative Commons Attribution 3.0 Unported License, https://creativecommons.org/licenses/by/3.0/). The rightmost panel of Figure 1 was adapted from [20] (© 2023 Y. Sasaki et al., distributed under the terms of the Creative Commons Attribution 4.0 International License, https://creativecommons.org/licenses/by/4.0).

Previous reviews have discussed chemosensors for metal ions [6–9], approaches for self-assembled chemosensors [17,19,31], chemosensor arrays (nanoparticles [32–33], polymers [34–35], and self-assembled elements [36]), solid-state analytical devices using gels [37–38] and paper substrates [21], and chemometric techniques [39] and their principles [40–41], independently. However, the conceptual connection between molecular-scale recognition events and device-level sensing architectures remains insufficiently explored. In this perspective, we propose a cross-scale design that bridges ion and molecular recognition chemistry with solid-state sensing devices incorporating nanoscale architectures for environmental monitoring of metal ions. This perspective introduces the principles of metal ion detection using chemosensors and the design strategy based on molecular self-assemblies for sensing multiple analytes based on pattern recognition [13,15]. After the discussion of the potential of self-assembled chemosensors and their arrays for simultaneous detection of metal ions in solution states, the concept is expanded to a paper-based chemosensor array device [20]. In this perspective, we introduce the new concept “cross-scale design”, defined as an approach connecting molecular sensing in solution states and solid-state sensing, while maintaining or enhancing chemosensor behaviors compared with those in solution (Figure 1). In this context, the concept of cross-scale design refers to the hierarchical integration of sensing processes across multiple levels, from supramolecular chemosensors and pattern-recognition-based sensing to device-level architectures for practical environmental monitoring.

## Discussion

### Designs and preparation of chemosensors for the simultaneous detection of metal ions

As described in the Introduction, we aim to simultaneously discriminate multiple metal ions based on pattern recognition. In pattern recognition-driven chemical sensors, various response patterns including absorbance, fluorescence intensities, and wavelength changes are used as input data for pattern recognition [36]. To perform accurate analysis, appropriate designs of chemosensors are required. This section focuses on the design strategies based on molecular self-assemblies and the candidates for building blocks for chemosensor elements.

#### Categorization of chemosensor designs

**Figure 2:**
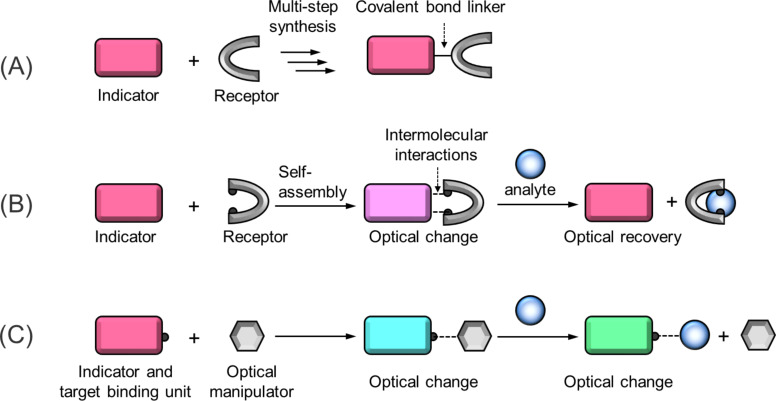
Categorization of chemosensor preparation and detection principles. (A) An indicator–spacer–receptor-based chemosensor based on covalent bond chemistry, (B) an indicator displacement assay for analyte capture based on a competitive reaction among the indicator, the receptor, and the analyte, and (C) a self-assembled chemosensing system based on a competitive reaction among the indicator (and target binding unit), the optical manipulator, and the analyte. Figure 2 was adapted from [17] (© 2023 Y. Sasaki & T. Minami. *ChemNanoMat* published by Wiley-VCH GmbH, distributed under the terms of the Creative Commons Attribution 4.0 International License, https://creativecommons.org/licenses/by/4.0).

In the simultaneous detection of multiple metal ions in aqueous media, chemosensors should satisfy the following requirements: They must exhibit different optical properties depending on the types of metal ions and their concentrations to obtain fingerprint-like response patterns, and they must be highly soluble [17]. Typical chemosensor molecules are covalently linked among the binding sites for analytes, optical units (chromophores and fluorophores), and manipulator units, if necessary (Figure 2A) [42]. To construct a chemosensor array, the variation of optical properties is significant, while the modification of chemosensor properties is limited by the synthetic effort required. Molecular self-assembly is a valid approach to spontaneously obtain an ensemble in aqueous media by mixing building blocks and to tune optical properties by changing the combination of building blocks [17,19,31].

One representative self-assembly-driven chemosensing system is an indicator-displacement assay (IDA) [31,43]. In this system, an indicator and a receptor unit spontaneously form a self-assembled chemosensor in aqueous media, accompanied by an optical change. Upon analyte addition, the indicator is released from the chemosensor ensemble, which induces optical recovery (Figure 2B). To further tune the optical properties of indicators, building blocks acting as optical manipulators are an appropriate strategy in self-assembled chemosensing. The chemosensor is formed using optical units (and the target binding site) and optical manipulators [13,15]. In contrast to the typical IDA, the approach using the optical unit serving as the target binding site and the optical manipulator induces distinct optical changes upon analyte capture (Figure 2C). This approach offers the advantage of facile preparation of chemosensors that display a variety of optical patterns, even with a small number of building blocks.

#### Selection of building blocks of chemosensors for metal ion sensing

The general strategy of metal-ion recognition primarily relies on multivalent coordination between target metal ions and donor atoms (e.g., nitrogen, oxygen, and sulfur) of binding sites [44]. Upon metal ion capture, the chemical information is visualized using optical units and further amplified by optical manipulators. To induce recognizable optical responses, molecular dynamics during the association and dissociation of color manipulators are significant [36].

Dynamic covalent bonds are key motifs that contribute to the formation of self-assembled chemosensors and analyte detection [45–46]. Phenylboronic acid derivatives have been widely used as the building blocks for versatile supramolecular architectures [47–49]. The inherent reversibility derived from dynamic covalent bonds endows unique functions to the self-assembled structures. In the field of analytical chemistry, beneficial molecular behavior based on boronate esterification has been applied to reversible and continuous analyte detection [48]. In conventional chemosensor designs, phenylboronic acid derivatives are widely used as receptor units for analytes with cis-diol moieties [47–49]. Also, the favorable binding affinity of phenylboronic acid derivatives enables the formation of ensembles with indicators bearing cis-diol units [50–52].

Catechol derivatives demonstrate favorable metal-ion recognition abilities, owing to the presence of two adjacent hydroxy groups [53]. The optical properties of catechol derivatives correspond to the protonation and deprotonation forms [54]. The coordination with metal ions promotes the deprotonation of catechol derivatives from neutral structures [53]. Herein, we focus on phenylboronic acid as an optical manipulator of catechol derivatives that act as indicators and binding sites for metal ions and discuss the usability of this combination in the simultaneous detection of metal ions.

### Dynamic covalent bond-based chemosensors for metal ion detection in solution states

**Figure 3:**
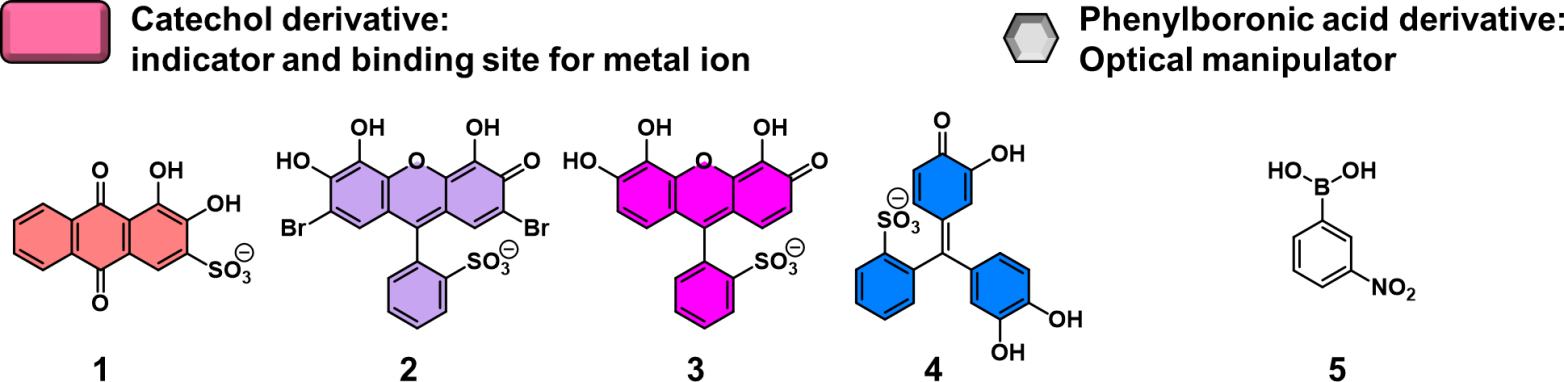
Chemical structures of catechols **1**–**4** and phenylboronic acid derivative **5**.

**Figure 4:**
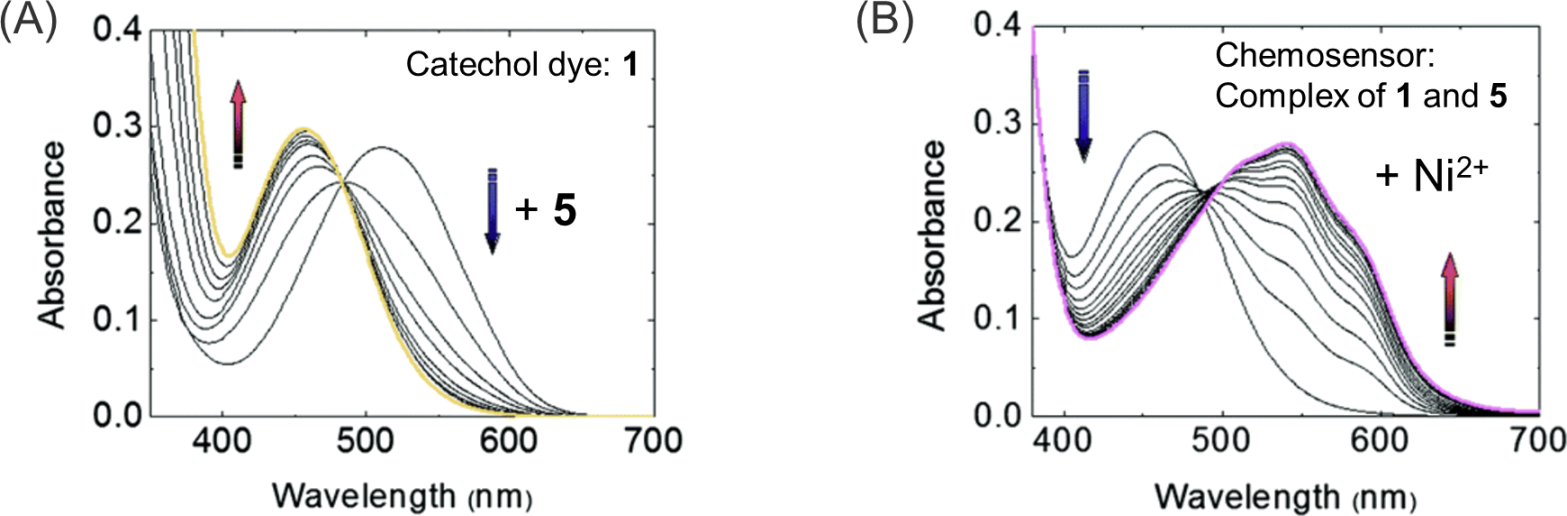
Colorimetric changes of a catechol dye **1** derived from (A) association with **5** through boronate esterification and (B) competitive coordination with Ni^2+^ in the presence of **5** in aqueous media. Figure 4, panels A and B were used with permission of The Royal Society of Chemistry, from [13] (“A molecular self-assembled colorimetric chemosensor array for simultaneous detection of metal ions in water” by Y. Sasaki et al., *Chem. Commun.*, Vol. 53, Issue 49, © 2017). This content is not subject to CC BY 4.0.

In this chemosensor design, catechol derivatives (i.e., alizarin red S (**1**), bromopyrogallol red (**2**), pyrogallol red (**3**), and pyrocatechol violet (**4**)) and a phenylboronic acid derivative (i.e., 3-nitrophenylboronic acid (**5**)) are employed (Figure 3) [13,20]. These building blocks are assembled through boronate esterification, which induces an optical change. For example, the catechol dye **1** showed blueshifts in the UV–vis absorption spectra with an increase in concentration of **5**, which supported the spontaneous formation of a boronate ester (Figure 4A). The nitrophenyl moiety acting as an electron-withdrawing group of **5** increases the Lewis acidity of boronic acid and facilitates the formation of boronate esters [11]. Meanwhile, the colorimetric ensemble of **1** and **5** exhibited redshifts in the UV–vis absorption spectra in the presence of a target metal ion (Ni^2+^). The optical changes suggested the coordination between **1** and the metal ion, along with the release of **5**, according to a competitive reaction among **1**, **5**, and the metal ion (Figure 4B). In this sensing approach, the catechol derivative served as the binding site for metal ions, resulting in a unique spectral profile. Consequently, this method induces versatile optical patterns beyond simple signal recovery and, thus, enables simultaneous discrimination of various metal ions even with a small number of chemosensor elements.

### Scheme of pattern recognition-driven chemical sensing

**Figure 5:**
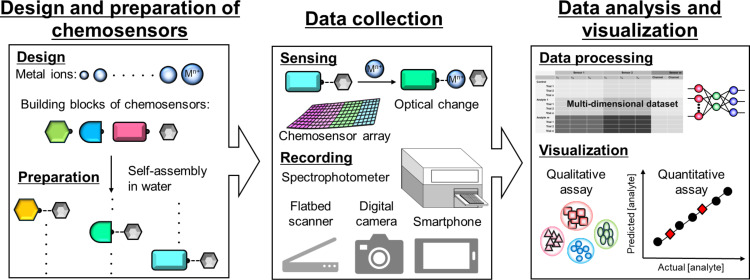
Conceptual scheme for pattern recognition-driven chemical sensing.

The process of simultaneous detection comprises several steps, namely, design and preparation of chemosensors, recording chemosensor responses to analytes, collection and preparation of input data, data processing using chemometric techniques, and visualization of chemical information (Figure 5) [36]. The optical responses of chemosensors and their arrays are acquired using spectrophotometers and portable recording apparatuses. The recorded optical responses as spectral profiles and digital images are summarized into a data matrix, according to the categories of types of chemosensor elements, analytes, and their concentrations. To validate the reproducibility of the sensing performance, replicate measurements are carried out. Therefore, the data matrix contains multidimensional chemical information, which can be processed using chemometric methods to classify and predict chemical information and to visualize the results as two- and three-dimensional outputs [39].

Herein, the assays are categorized into three types, namely, qualitative (i.e., discrimination among analyte types), semiquantitative (i.e., discrimination among analyte types and concentration levels), and quantitative detection (i.e., prediction of unknown analytes and their concentrations in real samples) [36]. Next, data processing is performed using supervised and unsupervised methods, depending on the purpose of the analysis. Unsupervised methods include principal component analysis (PCA) and hierarchical clustering analysis (HCA), while supervised methods include linear discriminant analysis (LDA), support vector machine (SVM), and artificial neural network (ANN). The details of each method have already been summarized in previous reviews [39]. In this perspective, simultaneous detection using LDA and SVM is introduced as an example [39]. LDA reduces the dimensionality of the input data and classifies each component based on its differences [39]. In the following sections, LDA is introduced for qualitative discrimination, whereas SVM is used for quantitative assays [55]. Thus, the analytical method can be used for quantitative assays in complicated media, such as mixtures of analytes and real samples in the presence of interfering species.

### Colorimetric chemosensor arrays for qualitative detection of eleven metal ions

**Figure 6:**
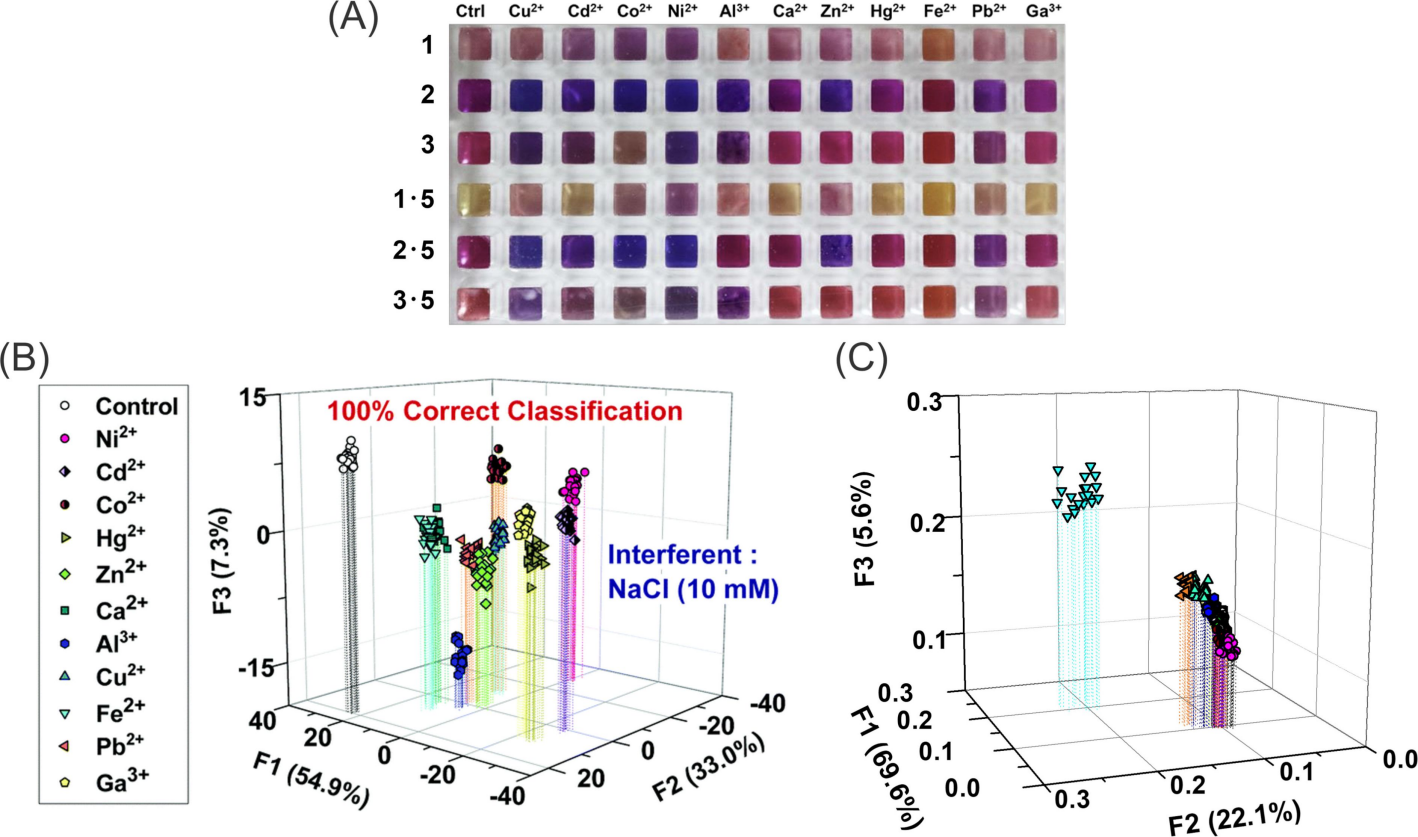
(A) Colorimetric patterns of a stand-alone dye array and the self-assembled chemosensor array in the presence or absence of metal ions. LDA canonical score plots for the qualitative assay of eleven metal ions using (B) the self-assembled chemosensor array and (C) the stand-alone dye array in aqueous media. Figure 6, panels A and B were used with permission of The Royal Society of Chemistry, from [13] (“A molecular self-assembled colorimetric chemosensor array for simultaneous detection of metal ions in water” by Y. Sasaki et al., *Chem. Commun.*, Vol. 53, Issue 49, © 2017). This content is not subject to CC BY 4.0. Figure 6, panel (C) was reproduced with permission from [56]. © 2018 Institute of Industrial Science, The University of Tokyo. This content is not subject to CC BY 4.0.

To explore the potential of colorimetric self-assembled chemosensors, the three catechol dyes **1**–**3** and the color manipulator **5** were selected as components of an array for the simultaneous discrimination of eleven types of metal ions (i.e., Pb^2+^, Cu^2+^, Zn^2+^, Ni^2+^, Cd^2+^, Co^2+^, Fe^2+^, Hg^2+^, Al^3+^, Ga^3+^, and Ca^2+^) [13]. As described in Figure 6A, the various colorimetric patterns were obtained on an array, according to the combination of the colorimetric ensembles and target metal ions. Importantly, the magnitude of the color changes of the catechol dyes in response to the metal ions was significant in the presence of **5**, unlike without the color manipulator (i.e., stand-alone dye array). Indeed, the qualitative detection with 100% correct classification was achieved for eleven types of metal ions, even with excess amounts of sodium chloride (NaCl) (Figure 6B). In this assay, the input data for LDA were constructed using UV–vis absorption spectra of chemosensors upon analyte addition. The dimensionality of the input data depends on the number of chemosensors, analytes, and replicate measurements, which is decreased using LDA. Each axis (i.e., factor, F) corresponds to the contribution derived from the multidimensional colorimetric response dataset [39]. The stand-alone dye array did not show accurate discrimination of the same metal ions (Figure 6C) [56]. Overall, the spontaneous preparation approaches using the catechol and the phenylboronic acid derivatives suggested the feasibility of simultaneous detection of metal ions in solution states, based on cross-reactivity and reversibility of self-assembled chemosensors.

### Solid-state chemosensor array device toward on-site metal ion detection

Based on the concept described above, a paper-based chemosensor array device was fabricated by printing self-assembled chemosensor elements onto paper substrates. To demonstrate the concept of cross-scale design, this section introduces a paper-based chemosensor array device embedded with the colorimetric ensembles that have already been evaluated in solution states. Herein, the key aspect of this concept is the retention of sensing performance of the self-assembled chemosensors even on the paper substrates.

#### Design and fabrication of the paper-based chemosensor array device

The uniformity of the sensing layer significantly affects the reproducibility and accuracy of analyte detection. Therefore, a printed pattern with 96 microwells was designed to avoid coffee ring effects [57]. Device designs (well design and area length and width), printing conditions and process (printing cycles of chemosensor inks), and sensing process (analyte solution volume) were optimized using a central composite design [58].

The selection of appropriate paper substrates also plays a significant role in reproducible sensing [59]. The mechanical properties of paper substrates depend on their thickness, pore size, and liquid absorbability [21]. In particular, the thickness of the substrates influences the magnitude of optical changes [60]. In addition, the fiber structures of paper substrates effectively contribute to the high dispersion of chemosensor elements [28].

**Figure 7:**
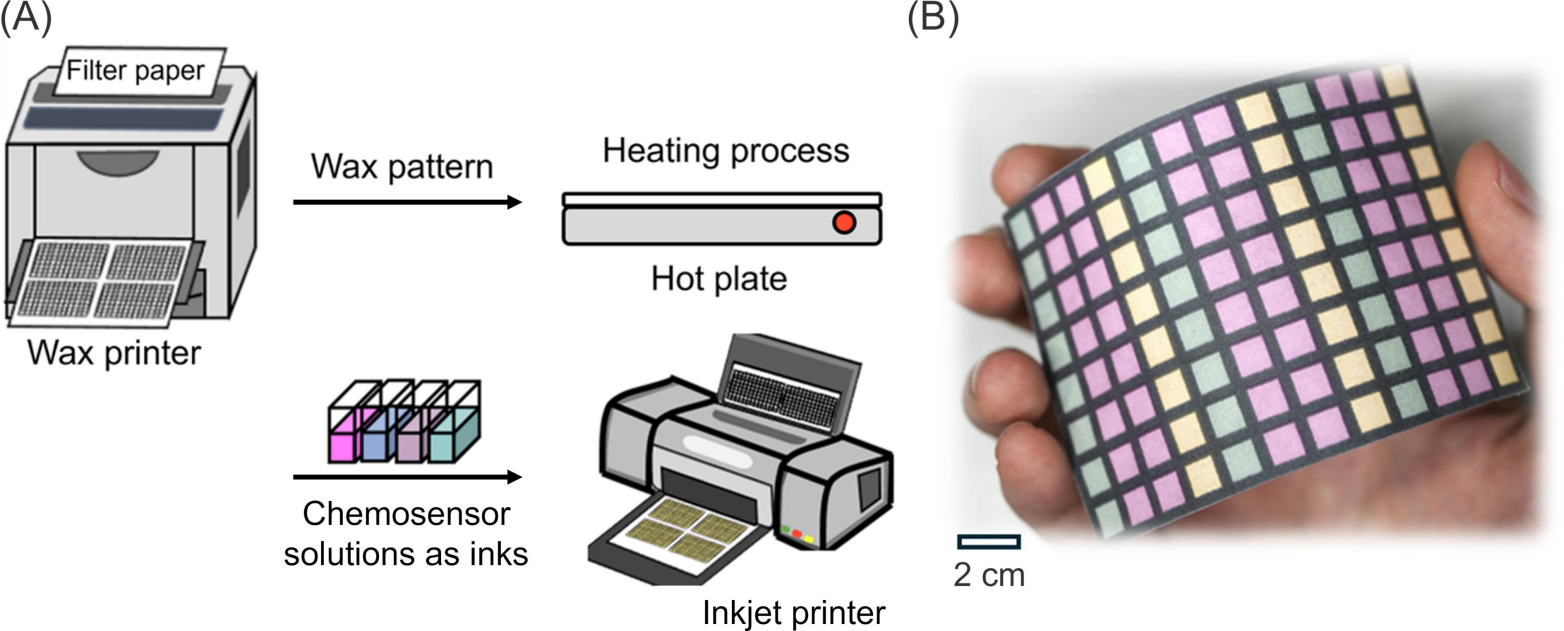
(A) Schematic of device fabrication using office printers and apparatus. (B) Photograph of the 96-well microtiter paper-based chemosensor array device. Figure 7 was adapted from [20] (© 2023 Y. Sasaki et al., distributed under the terms of the Creative Commons Attribution 4.0 International License, https://creativecommons.org/licenses/by/4.0).

The fabrication process in Figure 7A begins with patterning a hydrophobic layer using an office wax printer. The printed wax layer melts on a hot plate during annealing. Next, an inkjet printer is employed to deposit chemosensor solutions onto predefined regions of the paper substrate. The fiber structures of paper substrates contribute to highly dispersed chemosensor elements, resulting in uniform solid-state sensing layers (Figure 7B). Upon exposure to metal ions in aqueous solutions, the immobilized chemosensors interact with the analytes and induce optical changes on the paper substrate. These optical patterns are rapidly recorded using portable devices. Subsequently, the captured digital photographs are processed using imaging analysis to extract color information (e.g., RGB values) and further analyzed using pattern-recognition methods. Indeed, each chemosensor on the paper device showed different colorimetric responses to metal ions, indicating the fingerprint-like patterns containing multidimensional optical information.

#### Solid-state simultaneous detection of metal ions

**Figure 8:**
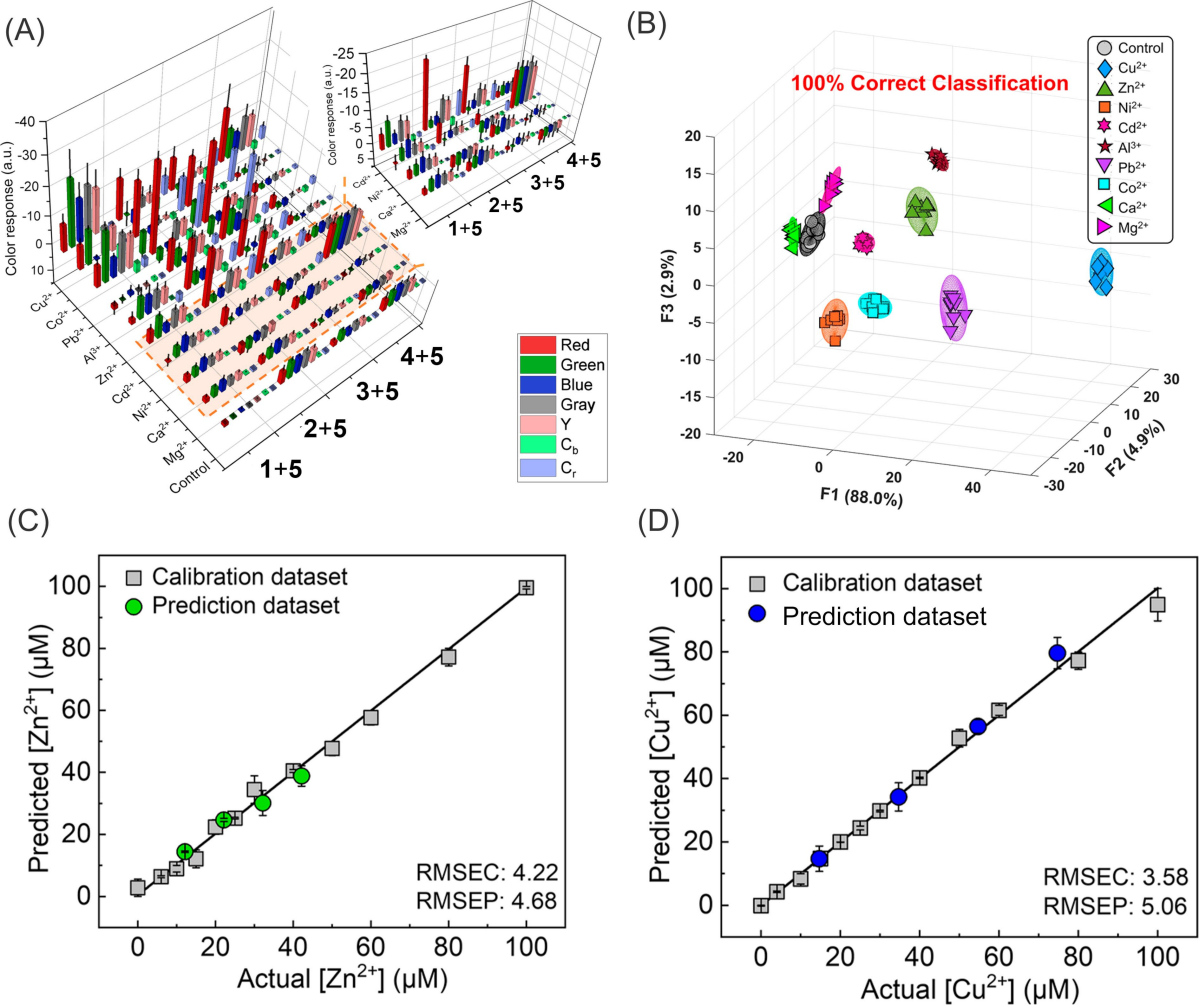
(A) Color response profile of the paper-based chemosensor array device upon adding target metal ions. (B) LDA canonical score plot for the qualitative assay of nine types of metal ions using the paper-based chemosensor array device. Results of the SVM regression for (C) Zn^2+^ and (D) Cu^2+^ in a river water sample. The values of the root-mean-square errors of calibration (RMSEC) and prediction (RMSEP) suggest the accuracy of the established model and prediction results. Figure 8, panels A–D were reproduced from [20] (© 2023 Y. Sasaki et al., distributed under the terms of the Creative Commons Attribution 4.0 International License, https://creativecommons.org/licenses/by/4.0).

Next, the 96-well microtiter paper-based chemosensor array device embedded with four types of colorimetric ensembles using building blocks **1**–**5** was employed for the qualitative discrimination of nine types of metal ions. Although the same sensor molecules were applied, the sensing environments are significantly different in solution and in the solid state. Therefore, in contrast to solution-based chemosensors, an additional ensemble made of **4** and **5** was included in the chemosensor library as an array component to increase optical response patterns. The obtained colorimetric changes on the device were rapidly recorded using a flatbed scanner [20]. The fabricated device demonstrated 100% correct classification of nine types of metal ions (Figure 8B). From the sensitivity aspect, the detectable ranges of the paper-based device were comparable to those of the solution-based chemosensor array. The concept can also be extended to polymer-based chemosensor elements. In a previous study, a polythiophene derivative immobilized chemosensor array on a paper substrate achieved highly sensitive detection of metal ions at tens of parts per billion levels, which was comparable to the detection range of an analytical method based on a stationary spectrophotometer [28].

#### Applicability of the paper-based chemosensor array to real-sample analysis

Herein, the usability of the paper-based chemosensor array device in real-sample analysis is introduced [20]. To realize the concept of cross-scale design, the sensor performance of chemosensor elements in solution states should be maintained or improved on the paper substrates. In environmental assessment, metal ion levels in river water indicate the state of ecological systems [1–2]. The colorimetric responses in the presence of interferents show non-linearity. Therefore, a powerful machine-learning method, SVM, was employed to build a linear calibration line from datasets constructed by color channels. Spike-and-recovery tests toward Zn^2+^ and Cu^2+^ demonstrated reliable analytical performance with recovery rates of 89–125%, even without sample purification (Figure 8C,D). To compare the sensing performance of the paper-based analytical device, recent paper-based colorimetric sensor devices for metal ion detection are summarized in Table 1. As shown, many paper-based analytical devices enable real-sample analysis with simple operation and minimal sample pretreatment. Indeed, the sensor performance from the viewpoint of usability and applicability is beyond that of solution-based chemosensors in metal ion sensing.

**Table 1:** Comparison of representative paper-based colorimetric sensing devices for metal-ion detection.

Probe/device	Medium	LOD (µM)^a^	Key metrics	Ref.

plug-and-play µPAD with colorimetric reagents (bathophenanthroline, dimethylglyoxime, and bathocuproine)	river water	Fe^2+^ 1.79, Ni^2+^ 5.11, Cu^2+^ 3.15	recovery rate (76–121%) agrees with AAS	[61]
inkjet-printed paper optode	drinking and environmental water	Pb^2+^ N.R.^b^; dynamic range 1–10	quick sensor response (<5 min)	[62]
mesoporous silica nanosphere-coated paper chemosensor	water	Cd^2+^ 0.22, Co^2+^ 0.28, Ni^2+^ 0.44, Fe^3+^ 0.54	reusability (for five cycles) after chelate treatment	[63]
silica nanoparticle-modified rhodamine B paper strip	drinking water	Cu^2+^ 11.0	sensor performance validated by ICP-OES	[64]
imidazole–dithiocarbamate derivative-coated tissue-paper strip	water samples	Cu^2+^ 0.0151, Hg^2+^ 1.17	different optical properties depending on the target metal ions	[65]
chemosensor array device functionalized with ensembles of catechol dyes and a phenylboronic acid derivative	river water	Cu^2+^ 2.36, Zn^2+^ 2.60, Ni^2+^ 4.77	qualitative and quantitative assays in metal-ion mixtures; recovery rate: 89–125% for Zn^2+^ and Cu^2+^ in river water	[20]
capillary-flow microfluidic paper device functionalized with colorimetric reagents (dimethylglyoxime, bathocuproine, and bathophenanthroline)	water	Ni^2+^ 34.1, Cu^2+^ 4.72, Fe^3+^ 19.7	recovery rates of 80–110% for >90% of collected samples	[66]
sodium rhodizonate-attached paper device	drinking, tap, and Ilam platform water samples	Ba^2+^ 26.5, Sr^2+^ 54.2	smartphone-assisted assay applying a masking strategy	[67]
bi-ligand cobalt-MOF nanozyme with 3,3′,5,5′-tetramethylbenzidine/H_2_O_2_ chromogenic system	environmental water	Cu^2+^ 0.130	smartphone-assisted sensor for on-site analysis	[68]
paper device immobilized with eriochrome black T (EBT) and bromothymol blue (BTB)	aqueous medium	Pb^2+^ (BTB) 29.0, Pb^2+^ (EBT) 86.9	high selectivity against other ions; thermal stability at 25–90 °C	[69]
dithizone colorimetric reagent immobilized on paper using a polyvinyl chloride matrix plasticized with dibutyl phthalate	water	Pb^2+^ 6.08, Cd^2+^ 0.890	rapid detection (150 s for Pb^2+^ and 60 s for Cd^2+^)	[70]
naphthylamine–quinolinol azo chemosensor-attached test strip	water and food samples^c^	Cd^2+^ 0.0262	reusability for six cycles with a chelate agent	[71]
dual-sided capillary microfluidic paper device immobilized with colorimetric reagents (dimethylglyoxime, bathocuproine, and bathophenanthroline)	river, tap, pond, drinking water	Ni^2+^ 22.1, Fe^3+^ 5.37, Cu^2+^ 3.15	recovery rate: 86–112%; stable for >4 weeks	[72]
paper strips using chromogens (cuprizone, 1,5-diphenylcarbazide, zincon monosodium salt, and 1-(2-pyridylazo)-2-naphthol)	environmental water	Cu^2+^ 4.72, Cr(VI) 5.00, Zn^2+^ 11.0, Mn^2+^ 5.10	comparable sensing performance with AAS	[73]

^a^LOD values were converted to “µM” for direct comparison. Reported ppm values were treated as “mg·L^−1^” for dilute aqueous samples. Atomic weights used for conversion were Fe 55.845, Co 58.933, Ni 58.693, Cu 63.546, Zn 65.38, Cd 112.414, Ba 137.327, Sr 87.62, Cr 51.996, Mn 54.938, Hg 200.59, and Pb 207.2 g·mol^−1^. For Cr(VI), conversion was based on the atomic weight of Cr. ^b^N.R.: not reported; the corresponding study reported a dynamic range rather than an explicit LOD. ^c^Food samples included potato, mushroom, apple, carrot, corn, barley, cashew, and sunflower seeds.

## Conclusion

In this perspective, we described the concept of cross-scale design for metal ion detection by integrating self-assembled chemosensors with solid-state analytical devices. Self-assembled chemosensor systems based on dynamic covalent bonds between catechol and phenylboronic acid derivatives provide a versatile platform for the spontaneous preparation of chemosensor elements without extensive synthetic effort. The reversible formation of boronate esters enables cross-reactive sensing behavior, providing fingerprint-like response patterns for various metal ions and their concentrations. By applying chemometric methods, multidimensional chemical information can be visualized qualitatively and quantitatively.

To address the challenges of metal ion analysis outside laboratories, paper-based platforms are attractive owing to their compatibility with office-printing technologies and portable imaging tools. Importantly, the paper-based chemosensor array device demonstrated reliable analytical performance in river water samples without the need for stationary analytical instruments or sample preparation.

The combination of molecular self-assembly, pattern-recognition sensing, and printable analytical devices provides a promising strategy for bridging molecular sensing systems in solution and solid-state analytical devices toward practical metal ion assessment. We believe that such cross-scale design approaches will contribute to on-site detection of chemical species in water environments across microscopic to macroscopic scales. More broadly, the cross-scale design strategy presented here may provide a general framework for bridging molecular recognition systems into practical sensing technologies that operate across molecular, nanoscale, and device levels.

## Data Availability

Data sharing is not applicable as no new data was generated or analyzed in this study.
